# Differences in hypertension between informal and formal areas of Ouagadougou, a sub-Saharan African city

**DOI:** 10.1186/1471-2458-14-893

**Published:** 2014-08-30

**Authors:** Boukaré Doulougou, Séni Kouanda, Clémentine Rossier, Abdramane Soura, Maria Victoria Zunzunegui

**Affiliations:** Département de Médecine Sociale et Préventive, École de santé publique, Université de Montréal, 850 Rue Saint Denis, 3ème étage, Bureau S03-806, Montréal, QC H2X 0A9 Canada; Institut de Recherche en Sciences de la Santé (IRSS), Ouagadougou, 03 BP 7192, Burkina Faso; Institut Supérieur des Sciences de la Population (ISSP), Université de Ouagadougou, Ouagadougou, 03 BP 7118, Burkina Faso; Institut d’Études Démographique et du parcours de vie (I-DEMO), Université de Genève, 40 Boulevard du pont d’Arve, 1211 Genève, Suisse; Institut de Recherche en Santé Publique de l’Université de Montréal (IRSPUM), Montréal, QC Canada; Centre de Recherche du Centre Hospitalier de l’Université de Montréal (CRCHUM), Montréal, QC Canada

**Keywords:** Hypertension, Prevalence, Risk factors, Adult population, Rural-to-urban migrants, Ouagadougou

## Abstract

**Background:**

Countries of sub-Saharan Africa are increasingly confronted with hypertension and urbanization is considered to favor its emergence. This study aims to assess the difference in the prevalence of hypertension between formal and informal urban areas of Ouagadougou and to determine the risk factors associated with hypertension in these urban populations of sub-Saharan Africa.

**Methods:**

A cross-sectional survey was conducted in 2010 on 2041 adults aged 18 years and older in formal and informal areas of Ouagadougou. Data was collected through personal interviews conducted at home. Blood pressure and anthropometric measurements were taken by trained interviewers. Logistic regressions were fitted to identify factors associated with hypertension.

**Results:**

The overall prevalence of hypertension was 18.6% (95% confidence interval [CI], 16.9-20.3) and its detection was 27.4% (95% CI, 22.9-31.9). Prevalence of hypertension in formal settings was 21.4% (95% CI, 19.0-23.8), significantly higher than prevalence in informal settings: 15.3% (95% CI, 13.0-17.6). However, this difference disappeared after adjusting for age. In addition to age, being an unmarried woman (odds ratio [OR] = 1.7; 95% CI, 1.1-2.4), recent rural-to-urban migration (OR = 1.8; 95% CI, 1.2-2.8), obesity (OR = 1.8; 95% CI, 1.1-3.1) and physical inactivity (OR = 1.9; 95% CI, 1.2-3.0), were independent risk factors for hypertension.

**Conclusions:**

Hypertension is common among the adult population of Ouagadougou but its detection is low. While there are no differences between formal and informal areas of the city, rural-to-urban migration emerges as an independent risk factor. Known risk factors as obesity and physical inactivity are confirmed while the vulnerability of unmarried women and rural-to-urban migrants maybe specific to this west African population.

## Background

Developing countries are undergoing the epidemiological transition with the emergence of non-communicable diseases [1, 2]. Among the factors contributing to this transition are the increase in life expectancy at birth, rapid unplanned urbanization with poor health habits (increased sedentary lifestyle, increased smoking and alcohol consumption) and the nutrition transition [3–5]. Of an estimated 36 million deaths from non-communicable diseases in the world in 2008, 48% were caused by cardiovascular disease, 80% took place in developing countries [5]. Hypertension is identified as the main risk factor for cardiovascular disease [6]. In African countries, the prevalence of hypertension in adult populations varies between 20-34% and seems to be increasing [7–9]. This prevalence is higher in urban than in rural areas [7, 10, 11]. Urbanization has been proposed as the main factor driving these differences and the increasing trends [5, 11].

In the cities of developing countries, formal areas characterized by adequate spatial planning and utilities (water, electricity, telephone, road infrastructure) and informal areas with no urban development could be frequently distinguished [12]. According to United Nations estimates, due to the accelerated urbanization, the absolute numbers of people living in slums in the low and middle-income countries rose from 760 million in 2000 to 863 million in 2012. Sub-Saharan Africa was the region with the largest proportion (62%) of urban population living in slums [12]. In spite of their importance, very little is known about hypertension prevalence in these informal areas.

In 2003, a cross-sectional study found hypertension prevalence of 23% among population aged 18 and over in Ouagadougou, but it did not report separately for informal and formal areas of the city and did not examine risk factors [13]. A recent study among population aged 35 years and over from Ouagadougou, reported a prevalence of 40% with differences in hypertension prevalence between formal and informal areas (comparative morbidity figure = 1.15; 95% CI, 0.99-1.34) [14]. But this study did not examine traditional risk factors as smoking, alcohol intake or chronic conditions neither differences between populations of rural and urban extraction. Our study therefore aims to assess the difference in the prevalence of hypertension between formal and informal urban areas of Ouagadougou and to identify the risk factors associated with hypertension in these urban populations of sub-Saharan Africa.

## Methods

### Study site

Burkina Faso is a developing country located in West Africa with its capital Ouagadougou. In 2006, the population of Ouagadougou was 1.5 million and is expected to reach 5.8 million by 2030 [15]. Since 2008, the Institut Supérieur des Sciences de la Population (ISSP) of the Université de Ouagadougou established a population observatory called Ouagadougou Health and Demographic Surveillance System in Burkina Faso (Ouaga HDSS) (http://www.issp.bf/opo/). In 2010, the Ouaga HDSS covered five neighborhoods (2 formal and 3 informal) at the northern periphery of Ouagadougou with around 80 000 residents living in 18 310 households [15]. Ouaga HDSS is affiliated to the International Network for the Demographic Evaluation of Populations and Their Health (INDEPTH), which included 49 observatories in 20 countries of Africa, Oceania and Asia (http://www.indepth-network.org).

### Data

Data come from the health survey carried out between February and August 2010 in Ouaga HDSS site. A sample of 1941 households were randomly selected and in each household every subject 15 years and older was invited to participate. A response rate of 87.5% was recorded at the household level. For this article, only adults 18 years and older will be included to facilitate comparison with previous research on hypertension prevalence.

Questions were administered face to face by trained interviewers using pocket PCs. The adult questionnaire included 8 sections (description of health, accidents and violence, depression, lifestyle (physical activity, smoking, alcohol consumption, nutrition), access to health services, chronic diseases, anthropometric measurements and measurement of blood pressure). Information on age, education, marital status, migration status, occupation of participants were informed by routine collection of the surveillance system. The questions were administrated in French and Moore (the main local language).

Out of the 2210 participants aged 18 years and older drawn for the survey, 169 participants (7.65%) were excluded from the present analysis because they were out of home during the survey. The final sample size for this work was 2041.

### Variables

Outcome: Blood pressure (BP) was measured on a single occasion at the house of the participant by the trained interviewer using a digital automatic sphygmomanometer (Omron 3) with an appropriate cuff size. Three BP measures were done at the beginning of the interview after at least 5 minutes’ rest, and three other BP measures were taken at the end of interview in seating position. BP was taken successively at least 30 seconds apart on the same arm. For analysis, we use the mean of the last two measurements [16]. Individuals with a systolic BP ≥ 140 mm Hg and/or diastolic BP ≥ 90 mm Hg, were considered to be hypertensive. Detection of hypertension was defined as self-reporting of any prior diagnosis of hypertension by a healthcare professional among those who had blood pressure readings suggesting hypertension.

#### Independent variables

Age, sex, marital status, place of residence, read/write capability and occupational status were inquired. Formal areas have cadastral organization with streets and public services such as electricity, tap water, telephone, school, sanitation. Informal areas are squatter settlements and have no cadastral organization or public services. Migration status was described by two variables: the duration of residence in Ouagadougou coded into 3 categories (native of Ouagadougou, migrants who reside for less than 10 years, migrants who reside for 10 years and over); the provenance of participant coded into 3 categories (Ouagadougou for those who were born there; those coming from outside of Ouagadougou; foreign for those coming from other countries). Chronic conditions were assessed by asking the participant whether he/she had ever been told by a health professional that he/she had chronic disease such as diabetes mellitus, chronic bronchitis or asthma, stroke, arthritis, gout, stomach ulcer, cancer, AIDS, heart problems, tuberculosis and epilepsy. Participants were classified as chronically ill (those who have at least one of the listed diseases) and non-chronically ill, otherwise.

Smoking status was assessed by indicating whether the participant smoked tobacco products at the time of the survey. Participant alcohol intake was assessed with items from WHO’s Alcohol Use Disorders Identification Test (AUDIT) [17]. The frequency of alcohol consumption during the last 12 months was asked and the responses were “never”, “once a month or less”, “two to four time per month”, “ two to three times per week”, “four to six time per week”, and “daily”. For the analysis, participants were classified in three groups: Never for “never” answer; Occasionally for the 3 answers “once a month or less”, “two to four time per month” and “ two to three times per week”; Frequently for the 2 answers “four to six time per week”, and “daily”.

Physical activity was assessed by asking participants how many days in the last week they practice physical activity (strenuous physical, labor, bicycling, walking) for at least 10 minutes. Participants were classified as: low for those who had carried out physical activity one day or those accounting for less than 2 hours/week; intense for those who carried out physical activity for at least 4 days/week and for more than seven hours; moderate for the remaining group.

Participant’s weight was measured to the nearest 0.01 kg using a digital scale. Height was assessed to the nearest 0.01 m using a wooden stadiometer. Body mass index (BMI) was calculated by dividing the weight (kg) by the square of the height (m^2^). The participant was classified as underweight when BMI < 18.5; normal weight when 18.5 ≤ BMI < 25; overweight when 25 ≤ BMI < 30; obese when BMI ≥ 30.

### Statistical analysis

The prevalence of hypertension was estimated according to each risk factor. Differences in prevalence of hypertension by levels of categorical risk factors were assessed by using Chi square tests. T-test was used for the comparison of means. In order to estimate adjusted odds ratios for each hypertension risk factor logistic regressions were fitted. All statistical tests were two-tailed and a *P*-value ≤ 0.05 was considered statistically significant. Given the known vulnerability of widows and separated women, sex stratified analyses of civil status by hypertension was conducted and a combined variable “sex by civil status” included in the logistic model taking as reference the group of married women. Lastly, to account for possible dependence of some observations taken in the same household (1973 individuals in 1940 households), generalized estimating equations were fitted producing results very similar to those of fitted logistic regressions without accounting for clustering. All statistical analyses were performed using IBM SPSS 20 for Windows.

### Ethics

The protocol of the health survey was approved by the Ethics Committee for Health Research of the Ministry of Health of Burkina Faso. Informed consent was obtained from all participants.

## Results

Of the 2041 subjects included in this analysis, 45.8% lived in informal settings, 23% were native of Ouagadougou, 64% came from outside of Ouagadougou of whom 90% came from rural areas. Table 1 shows the sex specific characteristics of participants. Both men and women, subjects in the informal setting were younger, tended to live more frequently in marriage and were less able to read and write compared to the formal setting. Informal area has more migrants under 10 years than formal area. Considering each sex, the presence of chronic conditions was not different between formal and informal areas, while overweight, obesity were more frequent in formal than in informal settings. Subjects of informal areas were more often smokers compared with formal settings (11.7% vs 6.8%; *P* < 0.001). Among men, those in formal settings consumed more alcohol and had less physical activity than those from informal settings.

**Table 1:** **Characteristics of the participants by sex and by residence, Ouagadougou**

	Women		Men		All	
	Informal	Formal	Informal	Formal	Informal	Formal
	(N = 500)	(N = 637)	(N = 434)	(N = 470)	(N = 934)	(N = 1107)
	n (%)	n (%)	n (%)	n (%)	n (%)	n (%)
Mean age, (SD)	39.9 (17.9)	43.9 (18.9)	41.0 (15.7)	45.7 (19.1)	40.4 (16.9)	44.7 (19.0)
	*P* Value^a^ < 0.001		*P* Value^a^ < 0.001		*P* Value^a^ < 0.001	
**Civil status**						
Married	352 (70.4)	368 (57.8)	348 (80.2)	311 (66.2)	700 (74.9)	679 (61.3)
Other	148 (29.6)	269 (42.2)	86 (19.8)	159 (33.8)	234 (25.1)	428 (38.7)
	*P* Value < 0.001		*P* Value < 0.001		*P* Value < 0.001	
**Read/Write**						
No	400 (80.0)	423 (66.4)	290 (66.8)	238 (50.6)	690 (73.9)	661 (59.7)
Yes	100 (20.0)	214 (33.6)	144 (33.2)	232 (49.4)	244 (26.1)	446 (40.3)
	*P* Value < 0.001		*P* Value < 0.001		*P* Value < 0.001	
**Occupational status** ^**b**^						
Employed	23 (4.8)	36 (6.0)	91 (21.6)	108 (24.4)	114 (12.7)	144 (13.9)
Independent	188 (39.6)	205 (34.3)	207 (49.0)	153 (34.6)	395 (44.0)	358 (34.5)
Unpaid job	264 (55.6)	356 (59.6)	124 (29.4)	181 (41.0)	388 (43.3)	537 (51.6)
	*P* Value = 0.18		*P* Value < 0.001		*P* Value < 0.001	
**Duration of residence in Ouagadougou**						
Native	91 (18.7)	170 (28.0)	96 (22.5)	122 (26.9)	187 (20.5)	292 (27.5)
< 10 years	203 (41.8)	122 (20.1)	145 (34.0)	59 (13.0)	348 (38.1)	181 (17.1)
≥10 years	192 (39.5)	315 (51.9)	186 (43.6)	272 (60.0)	378 (41.4)	587 (55.4)
	*P* Value < 0.001		*P* Value < 0.001		*P* Value < 0.001	
**Chronic conditions**						
No	408 (81.6)	521 (81.8)	366 (84.3)	395 (84.0)	774 (82.9)	916 (82.7)
Yes	92 (18.4)	116 (18.2)	68 (15.7)	75 (16.0)	160 (17.1)	191 (17.3)
	*P* Value = 0.93		*P* Value = 0.90		*P* Value = 0.94	
**Body Mass Index**						
<18.5	58 (11.6)	60 (9.4)	44 (10.1)	56 (11.9)	102 (10.9)	116 (10.5)
18.5 to 24.99	329 (65.8)	354 (55.6)	339 (78.1)	322 (68.5)	668 (71.5)	676 (61.1)
25.0 to 29.9	89 (17.8)	154 (24.2)	48 (11.1)	73 (15.5)	137 (14.7)	227 (20.5)
≥ 30	24 (4.8)	69 (10.8)	3 (0.7)	19 (4.0)	27 (2.9)	88 (7.9)
	*P* Value < 0.001		*P* Value = 0.001		*P* Value < 0.001	
**Smoking status**						
No	493 (98.6)	635 (99.7)	332 (76.5)	397 (84.5)	825 (88.3)	1032 (93.2)
Current smoker	7 (1.4)	2 (0.3)	102 (23.5)	73 (15.5)	109 (11.7)	75 (6.8)
	*P* Value = 0.05		*P* Value = 0.002		*P* Value < 0.001	
**Alcohol intake**						
Never	370 (74.0)	450 (70.6)	281 (64.7)	263 (56.0)	651 (69.7)	713 (64.4)
Occasionally	93 (18.6)	145 (22.8)	106 (24.4)	160 (34.0)	199 (21.3)	305 (27.6)
Frequently	37 (7.4)	42 (6.6)	47 (10.8)	47 (10.0)	84 (9.0)	89 (8.0)
	*P* Value = 0.22		*P* Value = 0.006		*P* Value = 0.005	
**Physical activity**						
Low	291 (58.2)	402 (63.1)	173 (39.9)	256 (54.5)	464 (49.7)	658 (59.4)
Moderate	159 (31.8)	188 (29.5)	166 (38.2)	135 (28.7)	325 (34.8)	323 (29.2)
Intense	50 (10.0)	47 (7.4)	95 (21.9)	79 (16.8)	145 (15.5)	126 (11.4)
	*P* Value = 0.15		*P* Value < 0.001		*P* Value < 0.001	

Abbreviation: SD, standard deviation.

^a^Obtained by T-test; other *P* Value were obtained by Χ^2^ test.

^b^Variable with missing values (65 among women and 40 among men).

*P*values for comparisons of participants from informal areas to those from formal areas.

**Figure 1:**
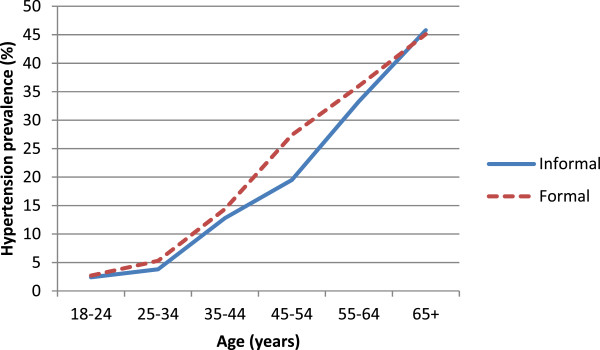
**Hypertension distribution by age and residence areas in Ouagadougou adult population.**

The prevalence of hypertension in the overall sample was 18.6% [95% confidence interval [CI], 16.9-20.3]. The formal setting had a prevalence of hypertension of 21.4% [95% CI, 19.0-23.8], while the prevalence in informal settings was 15.3% [95% CI, 13.0-17.6] and the difference was statistically significant. The weighted prevalence of hypertension was 10.4% [95% CI, 8.7-12.1] in informal areas and 16.7% [95% CI, 14.8-18.7] in formal areas. In both areas, not being able to read and write, coming from place outside of Ouagadougou and increasing age were associated with increased prevalence of hypertension (Table 2). The largest difference in the prevalence of hypertension between the areas occurred in middle age (Figure 1). In both informal and formal setting, the presence of self-reported chronic disease was not associated with hypertension and physical activity was associated with a decrease in prevalence. BMI and alcohol consumption were associated with hypertension, only in formal settings. Smoking was negatively associated with hypertension in informal settings but not in formal settings (Table 2).

**Table 2:** **Prevalence of hypertension by residence in adult population of Ouagadougou**

	Informal		Formal	
	N	% (95% CI)	N	% (95% CI)
All (unweighted)	934	15.3 (13.0-17.6)	1107	21.4 (19.0-23.8)
All (weighted)	1277	10.4 (8.7-12.1)	1386	16.7 (14.8-18.7)
**Sex**				
Female	500	16.4 (13.1-19.7)	637	20.4 (17.3-23.6)
Male	434	14.1 (10.8-17.3)	470	22.8 (19.0-26.6)
	*P* Value = 0.32		*P* Value = 0.35	
**Age, y**				
18-24	168	2.4 (0.05-4.7)	220	2.7 (0.6-4.9)
25-34	289	3.8 (1.6-6.0)	209	5.3 (2.2-8.3)
35-44	133	12.8 (7.0-18.5)	118	14.4 (8.0-20.8)
45-54	123	19.5 (12.4-26.6)	175	27.4 (20.8-34.1)
55-64	114	33.3 (24.6-42.1)	203	36.0 (29.3-42.6)
65+	107	45.8 (36.2-55.4)	182	45.1 (37.8-52.3)
	*P* Value < 0.001		*P* Value < 0.001	
**Civil status**				
Married	700	12.7 (10.2-15.2)	679	22.4 (19.2-25.5)
Other	234	23.1 (17.6-28.5)	428	19.9 (16.1-23.7)
	*P* Value < 0.001		*P* Value = 0.32	
**Read/Write**				
No	690	18.0 (15.1-20.8)	661	27.2 (23.8-30.6)
Yes	244	7.8 (4.4-11.2)	446	12.8 (9.7-15.9)
	*P* Value < 0.001		*P* Value < 0.001	
**Occupational status** ^**a**^				
Employed	114	7.9 (2.9-12.9)	144	17.4 (11.1-23.6)
Independent	395	15.2 (11.6-18.7)	358	24.9 (20.4-29.4)
Unpaid job	388	16.8 (13.0-20.5)	537	21.2 (17.8-24.7)
	*P* Value = 0.07		*P* Value = 0.16	
**Duration of residence in Ouagadougou** ^**a**^				
Native	187	10.7 (6.2-15.2)	292	11.3 (7.7-15.0)
< 10 years	348	14.4 (10.7-18.1)	181	17.1 (11.6-22.7)
≥ 10 years	378	18.0 (14.1-21.9)	587	27.9 (24.3-31.6)
	*P* Value = 0.07		*P* Value < 0.001	
**Provenance**				
Ouagadougou	187	10.7 (6.2-15.2)	292	11.3 (7.7-15.0)
Outside of Ouagadougou	658	17.3 (14.4-20.2)	649	27.4 (24.0-30.9)
Foreign	68	5.9 (0.1-11.6)	119	14.3 (7.9-20.7)
	*P* Value = 0.007		*P* Value < 0.001	
**Chronic conditions**				
No	774	15.9 (13.3-18.5)	916	20.7 (18.1-23.4)
One or more	160	12.5 (7.3-17.7)	191	24.6 (18.4-30.8)
	*P* Value = 0.28		*P* Value = 0.24	
**Body Mass Index**				
<18.5	102	19.6 (11.7-27.4)	116	31.9 (23.3-40.5)
18.5 to 24.99	668	14.8 (12.1-17.5)	676	17.8 (14.9-20.6)
25.0 to 29.9	137	14.6 (8.6-20.6)	227	23.8 (18.2-29.4)
≥ 30	27	14.8 (0.5-29.1)	88	29.5 (19.8-39.3)
	*P* Value = 0.65		*P* Value = 0.001	
**Smoking status**				
No	825	16.4 (13.8-18.9)	1032	21.8 (19.3-24.3)
Current smoker	109	7.3 (2.4-12.3)	75	16.0 (7.5-24.5)
	*P* Value = 0.01		*P* Value = 0.24	
**Alcohol intake**				
Never	651	15.5 (12.7-18.3)	713	22.3 (19.2-25.4)
Occasionally	199	11.6 (7.1-16.0)	305	15.1 (11.0-19.1)
Frequently	84	22.6 (13.5-31.8)	89	36.0 (25.8-46.1)
	*P* Value = 0.06		*P* Value < 0.001	
**Physical activity**				
Low	464	19.6 (16.0-23.2)	658	24.5 (21.2-27.8)
Moderate	325	10.8 (7.4-14.2)	323	19.5 (15.2-23.9)
Intense	145	11.7 (6.4-17.0)	126	10.3 (4.9-15.7)
	*P* Value = 0.001		*P* Value = 0.001	

Abbreviation: CI, confident interval.

^a^Sample sizes do not add to total because information was not available for all participants for these variables.

Given the lack of significant difference in hypertension prevalence between the formal and informal areas we fitted a multivariate logistic model to the whole sample to assess the significance of independent risk factors in both areas. As expected there was a significant gradient of higher hypertension prevalence with increasing age. Unmarried women were more likely to be hypertensive than married women and no differences were observed between married women and men, after adjustment for all remaining covariates in the model. Recent migrants to Ouagadougou (those arriving in the last 10 years) were more likely to be hypertensive than those born in Ouagadougou while the odds of being hypertensive did not differ between the more established migrants (more than 10 years) and the native population. As expected, obese people and those with low physical activity were more likely to be hypertensive while those who drank occasionally had lower odds of hypertension than those who never drank (Table 3).

**Table 3:** **Hypertension associated factors and corresponding multivariate odds ratios in Ouagadougou adult population** ^**a**^

Characteristics	N (% HT)	Unadjusted		Adjusted	
		OR (95% CI)	***P***value	OR (95% CI)	***P***value
**Age, y**					
18-24	368 (2.7)	1		1	
25-34	485 (4.1)	1.5 (0.7-3.3)	0.27	1.7 (0.8-3.7)	0.21
35-44	245 (13.9)	5.8 (2.8-11.9)	< 0.001	6.2 (2.9-13.3)	< 0.001
45-54	291 (24.4)	11.6 (5.8-22.9)	< 0.001	11.1 (5.3-23.1)	< 0.001
55-64	310 (35.5)	19.7 (10.1-38.5)	< 0.001	18.5 (9.0-38.1)	< 0.001
65+	274 (44.2)	28.3 (14.5-55.4)	< 0.001	22.8 (10.9-47.6)	< 0.001
**Civil status by sex**					
Married women	699 (12.7)	1		1	
Unmarried women	394 (29.4)	2.9 (2.1-3.9)	< 0.001	1.7 (1.1-2.4)	0.007
Married men	648 (22.5)	2.0 (1.5-2.7)	< 0.001	1.3 (0.9-1.8)	0.13
Unmarried men	232 (6.5)	0.5 (0.3-0.8)	0.01	1.1 (0.6-2.1)	0.85
**Read/Write**					
Yes	687 (10.8)	1		1	
No	1286 (22.7)	2.4 (1.9-3.2)	< 0.001	1.1 (0.8-1.5)	0.66
**Residence**					
Informal	913 (15.1)	1		1	
Formal	1060 (21.5)	1.5 (1.2-1.9)	< 0.001	1.2 (0.9-1.6)	0.16
**Duration of residence in Ouagadougou**					
Native	479 (11.1)	1		1	
< 10 years	529 (15.3)	1.5 (1.0-2.1)	0.05	1.8 (1.2-2.8)	0.006
≥ 10 years	965 (24.0)	2.5 (1.8-3.5)	< 0.001	1.4 (0.9-1.9)	0.10
**Body Mass Index**					
<18.5	211 (26.1)	1.8 (1.3-2.6)	0.001	1.0 (0.7-1.5)	0.90
18.5 to 24.99	1300 (16.2)	1		1	
25.0 to 29.9	353 (20.4)	1.3 (1.0-1.8)	0.07	1.4 (1.0-2.0)	0.05
≥ 30	109 (25.7)	1.8 (1.1-2.9)	0.01	1.8 (1.1-3.1)	0.02
**Smoking status**					
No	1793 (19.4)	1		1	
Current smoker	180 (10.6)	0.5 (0.3-0.8)	0.004	1.2 (0.7-2.0)	0.61
**Alcohol intake**					
Never	1316 (19.0)	1		1	
Occasionally	490 (13.7)	0.7 (0.5-0.9)	0.008	0.7 (0.5-1.0)	0.05
Frequently	167 (29.3)	1.8 (1.2-2.5)	0.002	1.1 (0.7-1.6)	0.69
**Physical activity**					
Intense	259 (11.2)	1		1	
Moderate	623 (15.1)	1.4 (0.9-2.2)	0.13	1.3 (0.8-2.1)	0.29
Low	1091 (22.3)	2.3 (1.5-3.4)	< 0.001	1.9 (1.2-3.0)	0.005

Abbreviation: OR, odds ratio; CI, confident interval; HT, arterial hypertension.

^a^adjusted model was fit on the complete data set ( N = 1973 participants) and took the clustering at household level into account.

*P*Values were obtained by Wald tests.

Among participants with elevated blood pressure readings suggestive of hypertension, the prevalence of detection was 27.4% (95% CI, 22.9-31.9). The proportion of these hypertensive participants aware of their condition was higher in formal setting than in informal setting (31.2% versus 21.0%; *P* = 0.03), but no difference was observed according to sex.

## Discussion

Our study shows that in the city of Ouagadougou, hypertension is common. There is no significant different in prevalence between informal and formal areas after age adjustment. Area specific analyses showed that factors such as older age, illiteracy, coming from an area of Burkina outside of Ouagadougou, physical inactivity are found as risk factors for hypertension in both formal and informal areas. In formal areas, those undernourished or with frequent alcohol consumption are more likely to be hypertensive. Among women, being unmarried was associated with hypertension.

The overall prevalence found in this study (18.6%) is somewhat lower than prevalence of previous studies conducted in the same population: For the same age group (18 years and over), a previous study reported 23% [13]. Considering participant aged 35 years and over, our results show a prevalence of 30.1% (95% CI, 27.5-32.8) less than 40% found in 2007 [14]. This does not mean that hypertension in Ouagadougou is declining. A reason for this conflictive results could be that in the current study no information on hypertensive medication was available. Despite our lower estimate, this study confirms that hypertension prevalence remains higher in the urban areas than in semi-urban and rural areas in Burkina [11]. However, the hypertension prevalence in the current study was lower that prevalence found in other African urban population aged 16 and over that showed prevalence between 30 to 41% [8, 18–20].

The lack of significant difference in age adjusted hypertension prevalence between the informal areas compared to formal areas of this study corroborates the results of Niakara among people aged 35 years and over [14], which found no significant age-adjusted difference between these two types of urban areas of Ouagadougou.

Associations of hypertension with older age, high BMI and physical inactivity corroborate previous results [14, 18, 21]. Both high BMI and physical inactivity are consequences of urbanization.

Migration from sub-Saharan African countries to western countries was identified as a risk factor of hypertension [22]. Our study shows that rural-to-urban within-country migration is positively associated with hypertension. Comparing the results of this study to our previous study in rural and semi-urban areas in the region of Kaya (Burkina Faso) and for the same age group, the prevalence of hypertension among migrants in Ouagadougou is higher than in rural areas [11]. Explanatory factors for this positive association could be the same as those found in international migration: anxiety and stress first, then changes in diet and physical inactivity [23]. Unwin also showed that after 12 months of migration from rural to urban areas, physical activity was significantly lowered and fat intake was increased among migrants in Tanzania [24] and that after the first twelve months of migration, blood pressure among migrants fell in both men and women [24]. This drop in blood pressure could be followed later by its elevation consequence of the poor health behaviors which are gradually acquired. Our finding that migrants under 10 years have higher odds of hypertension even after extensive covariates is contrary to those found in Dakar in Senegal, where a lower prevalence of hypertension was found in recent migrants [25]. This difference may be due to the origin of the migrants which were almost rural in our study while in Dakar, they mostly come from other cities in Senegal [25]. So the urbanization contrast between the areas they are coming from and the host areas could explain this difference.

After adjustment, there was no significant difference between migrants over 10 years (63% of those who have migrated more than 10 years ago had been in Ouagadougou more than 20 years) and natives reflecting the fact that after a long time of exposure to the city of Ouagadougou, hypertension patterns in immigrants converge with those of the natives, probably due to similar exposures to the city, similar health behavior and stress, similar opportunities of diagnosis and treatment and similar survival patterns. Also it is likely that rural-to-urban immigrants over 10 years arrived at a time when the city was smaller with consequent less stressful transitions from rural to urban contexts.

Unmarried people have higher prevalence of hypertension. This is consistent with our previous results in the semi-urban of Kaya, in the north central of Burkina Faso [11] but the current study has further identified that the higher prevalence of hypertension in unmarried people is restricted to unmarried women, meaning divorced (4%) and widowed (61%). Being widow is rare among African men, for whom re-marriage and polygamy are the social norm. Thus, our findings lead us to suggest that women who are widows are particularly vulnerable for hypertension in Burkina Faso compared to married women of similar age, education and health behaviors. Authors have also previously shown the high vulnerability of widows [26].

Moderate alcohol consumption was associated with a lower prevalence of hypertension even after extensive adjusting. This protective effect of light-to-moderate alcohol consumption has been shown by previous research in developed countries [27]. Even more, contrary to other studies, frequent alcohol consumption (4 times and over/week) was not significantly associated with hypertension but misclassification bias could hinder detection of an elevated odds ratio in the highest category of alcohol consumption. Different methods to quantify alcohol consumption make direct comparisons with previous studies difficult. However Pires et al. found a borderline association between hypertension and frequent alcohol consumption ( more than 3 days/week) in men with an odds ratio [OR] of 1.4 (95% CI, 0.9-2.2, *P* = 0.08) [28]. Future studies to better describe the problem in the African context are desirable.

Smoking appears to be a protective factor for hypertension in the informal environment; this negative association of smoking with hypertension was found in previous studies in Ghana (OR = 0.3; 95% CI, 0.1-0.7) [10]. However, this finding is confounded by age since smoking was more frequent in the younger population and, as expected, this association disappears in the age adjusted analysis as in other studies conducted in Africa [29, 30].

Our study shows that about one quarter of hypertensive adults knew their condition and that corroborates with previous results (30%) on the same population of Ouagadougou [31]. This proportion is low compared to that reported in Ghana (34.0%) [18]. Also, treatment of hypertension among adult population of Ouagadougou remains low (less than half hypertensive adults) according to that previous study [31]. Mass prevention by acting on risk factors should be a priority in order to shift the distribution of risk factors to lower levels of risk [32]. In addition, detection of hypertensive people in the community and clinical management of hypertension should improve. The modifiable risk factors identified in this study (BMI, physical activity) are aspects that need to focus, without forgetting other aspect as lifestyle and dietary habits [33]. Special surveillance of risk groups such as rural-to-urban migrants and widows are also very important in prevention. This prevention could be translated into actions through awareness and screening campaigns for hypertension, already suggested by other authors in the African context [34].

The main limitation of this study was the lack of data on medication. This lack of information hinder our ability to identify hypertensive people under control of BP. This would entail an underestimation of prevalence. Estimates on the proportion of hypertensive people achieving controlled BP varies (between 3 and 16%) in African studies [7, 20]. Thus, the true prevalence in our sample would be underestimated by less than 10% of the true value.

## Conclusions

Our findings confirm that hypertension is common in Ouagadougou. Prevalence appears to remain stable in the last decade. There was no significant difference in prevalence between the informal and formal settings, once differences in age distribution were taken into account. Rural-to-urban migrants and unmarried women emerged as vulnerable populations for hypertension in these urban West African setting. Efforts should be made to reinforce healthy behaviors, particularly keeping a healthy body weight and regular physical activity, to control the emergence of cardiovascular diseases. In addition, the vulnerability of unmarried women should be recognized and targeted with gender equality policies.
